# Detection of a novel SME-6 Carbapenemase in *Serratia ureilytica* in Germany

**DOI:** 10.1093/jac/dkaf121

**Published:** 2025-04-12

**Authors:** Kaan Kocer, Lisa Göpel, Sarah Gisch, Leif Tueffers, Susanne Hauswaldt, Jan Rupp, Sébastien Boutin, Dennis Nurjadi

**Affiliations:** Institute of Medical Microbiology, University Hospital Schleswig-Holstein Campus Lübeck and University of Lübeck, Ratzeburger Allee 160, 23562 Lübeck, Germany; German Center for Infection Research (DZIF), Partner Site Hamburg-Lübeck-Borstel-Riems, Lübeck, Germany; Institute of Medical Microbiology, University Hospital Schleswig-Holstein Campus Lübeck and University of Lübeck, Ratzeburger Allee 160, 23562 Lübeck, Germany; Institute of Medical Microbiology, University Hospital Schleswig-Holstein Campus Lübeck and University of Lübeck, Ratzeburger Allee 160, 23562 Lübeck, Germany; Institute of Medical Microbiology, University Hospital Schleswig-Holstein Campus Lübeck and University of Lübeck, Ratzeburger Allee 160, 23562 Lübeck, Germany; Institute of Medical Microbiology, University Hospital Schleswig-Holstein Campus Lübeck and University of Lübeck, Ratzeburger Allee 160, 23562 Lübeck, Germany; Institute of Medical Microbiology, University Hospital Schleswig-Holstein Campus Lübeck and University of Lübeck, Ratzeburger Allee 160, 23562 Lübeck, Germany; German Center for Infection Research (DZIF), Partner Site Hamburg-Lübeck-Borstel-Riems, Lübeck, Germany; Infectious Disease Clinic, University Hospital Schleswig-Holstein Campus Lübeck and University of Lübeck, Lübeck, Germany; Institute of Medical Microbiology, University Hospital Schleswig-Holstein Campus Lübeck and University of Lübeck, Ratzeburger Allee 160, 23562 Lübeck, Germany; German Center for Infection Research (DZIF), Partner Site Hamburg-Lübeck-Borstel-Riems, Lübeck, Germany; Airway Research Center North (ARCN), German Center for Lung Research (DZL), Lübeck, Germany; Institute of Medical Microbiology, University Hospital Schleswig-Holstein Campus Lübeck and University of Lübeck, Ratzeburger Allee 160, 23562 Lübeck, Germany; German Center for Infection Research (DZIF), Partner Site Hamburg-Lübeck-Borstel-Riems, Lübeck, Germany

## Abstract

**Background:**

Carbapenem resistance in *Serratia* species is occasionally mediated by the serine carbapenemase *Serratia marcescens* enzymes (SMEs). During microbiological diagnostics, we identified a carbapenem-resistant *Serratia ureilytica* isolate in which resistance was not mediated by any known SME variants or other characterized carbapenemases.

**Objectives:**

To identify and characterize the underlying resistance mechanism in a carbapenem-resistant *Serratia ureilytica* isolate that lacks known SME variants or other characterized carbapenemases.

**Methods:**

Antimicrobial susceptibility testing (AST) was performed using Vitek, gradient strips, broth microdilution, and disc diffusion methods. WGS was performed to identify the resistance mechanisms. Growth curve analysis and RT-qPCR were performed at 30°C and 37°C.

**Results:**

WGS identified a novel SME variant, SME-6, which differed from a known variant, SME-2, by two amino acids (G117R and G147E). AST showed carbapenem resistance at 30°C but susceptibility at 37°C. Growth curve analysis showed a shorter lag phase at 30°C compared with 37°C, and RT-qPCR showed a ∼3-fold higher *bla*_SME_ expression at 30°C.

**Conclusions:**

To the best of our knowledge, this study reports the first identification of SME-6 and the first detection of an SME-type carbapenemase in Germany. Resistance was found to be temperature-dependent, with faster growth and higher SME-6 expression at lower temperatures contributing to the phenotype. These findings suggest SME variants may be underdiagnosed using current diagnostic protocols, highlighting the need for adjustments to improve detection of temperature-sensitive resistance mechanisms.

## Introduction

*Serratia marcescens* is an opportunistic pathogen capable of causing a range of infections, particularly in immunocompromised individuals. These infections can vary from relatively common conditions, such as urinary tract infections, to more severe diseases, including pneumonia, endocarditis, and wound infections.^1^ The emergence and rapid dissemination of carbapenemase-producing Enterobacterales represent a major global health concern.^1,2^ Among these*, Serratia marcescens* enzymes (SMEs) have been reported to confer carbapenem resistance in *Serratia* spp. SMEs are chromosomally encoded Ambler class A serine carbapenemases exclusive to *Serratia* spp.

Although these enzymes typically confer resistance to carbapenems, SME-producing *Serratia* spp. remain susceptible to extended-spectrum cephalosporins and are inhibited by both classical inhibitors, such as clavulanic acid, and newer beta-lactamase inhibitors.^3^ Since the first detection of SME in England in 1982,^4,5^ five variants (SME-1–5) have been described globally.^6–8^ Reports of SME-producing isolates are infrequent and usually limited to small outbreaks or clusters.^9^

In routine microbiological diagnostics, we identified a *Serratia marcescens* isolate (identified using MALDI-TOF mass spectrometry) displaying resistance to imipenem and meropenem. While reduced susceptibility to imipenem can occur in *Serratia* species in the absence of carbapenemases,^10,11^ resistance to meropenem is often mediated by carbapenemases.^10,11^ A positive result from the *in vitro* meropenem disk inactivation assay indicated the presence of a carbapenemase, prompting molecular characterization via whole-genome sequencing. Here, we report the detection of a novel SME variant, SME-6, in a *Serratia ureilytica* isolate (strain X47) recovered from the sputum of a hospitalized patient in Germany.

## Methods

### Patient data, routine microbiological diagnostics and additional antimicrobial susceptibility testing

The SME-6-harbouring *S. ureilytica* was isolated from a sputum specimen of a 76-year-old male patient in July 2024. Species identification was performed using MALDI-TOF MS (Bruker, Germany) and antimicrobial susceptibility testing (AST) was determined by the Vitek2 System (Biomérieux, Germany), with interpretation according to EUCAST clinical breakpoints version 14.0. Resistance was further tested using MIC gradient strips (Liofilchem, Italy). Molecular diagnostic for carbapenemase detection was performed using the isothermal amplification method (eazyplex^®^ SuperBug Complete A), which can detect VIM, NDM, KPC, OXA-48-like, OXA-23, OXA-40 and OXA-58. In addition, meropenem hydrolysis was investigated by the carbapenem disk inactivation method (CIM), as described previously.^12^ To confirm routine diagnostic findings, the frozen isolate was recultured and subjected to additional AST methods, including disc diffusion on Mueller–Hinton agar (bioMérieux) and broth microdilution (BMD) (MICRONAUT MDRO panel, Merlin, Bornheim-Hesel, Germany), following the manufacturer’s instructions. Meropenem-vaborbactam susceptibility was determined using Sensititre^TM^ BMD panel EUMDRXXF (Thermo Scientific^TM^, USA).

### Genome sequencing and bioinformatics analysis

WGS was performed using the Illumina Nextseq 2000 platform (2× 51 bp). Raw sequences were controlled for quality and adapter removal using fastp (v0·23·2 with parameters -q = 30 and -l = 45).^13^ Clean reads were then used to create *de novo* assembly using SPAdes 3.15.5 (with the option —careful and—only-assembler).^14^ Draft genomes were curated by removing contigs with a length <500 bp and/or coverage <10×. The quality of the final draft was quality-controlled using Quast (v5.0.2).^15^ The genome was compared with 254 publicly available genomes from the *Serratia* genus from NCBI Microbial Genome Resources. Each genome was annotated using pgap (version 2023-10-03.build7061) and the core genome was evaluated using Roary (v 3.13.0).^16^ 2118 genes present in all isolates were used to build a phylogenetic tree. In addition, the species identification of each genome was done using mash (sub-command screen) by screening each genome to a database composed of a representative genome of each species present in the Microbial Genomes resource (https://www.ncbi.nlm.nih.gov/genome/microbes/). Antimicrobial resistance genes presence were screened using Abricate (minimum identity 90% and minimum coverage 80%) (https://github.com/tseemann/abricate) to identify antimicrobial resistance (NCBI, CARD, ARG-ANNOT, ResFinder, MEGARES databases).^17–22^

### Growth analysis

Growth curves were performed on three biological replicates in antibiotic-free Mueller–Hinton broth (MHB) at 37°C and 30°C. The growth curves were initiated with a 1:100 dilution of 0.5 Mcfarland suspension prepared from overnight cultures (∼18–20 h incubation at 37°C and 30°C). Growth was monitored at 37°C for cultures incubated at 37°C and 30°C for cultures incubated at 30°C, using a 96-well microplate and a BioTek Epoch 2 plate reader (Agilent Technologies, USA). Optical density at 590 nm (OD590) was measured every 15 minutes. Data were visualized using GraphPad Prism v5.0 (GraphPad Software, La Jolla, CA, USA).

### RT–quantitative PCR analysis of bla_SME-6_ expression

Overnight cultures grown in MHB at 37°C and 30°C were diluted 1:100 in fresh antibiotic-free MHB to a total volume of 2 mL and allowed to grow to mid-exponential growth phase (OD_600_∼0.5) at the respective temperatures. RNA was extracted using the NucleoSpin^™^ RNA Kit (Macherey-Nagel, Germany) and *bla*_SME_ expression was quantified relative to 16S rRNA using the Luna Universal Probe One-Step RT–qPCR kit (New England Biolabs, USA). The primers used were as follows: *bla*_SME_-fw 5′-TGACACTTCAACGCCAAAAG-3′, *bla*_SME_-rv 5′-AACCCAATCAGCAGGAACAC-3′, 16S-fw 5′-GCTAATACCGCATAACGTCG-3′, and 16S-rv 5′-TCATCCTCTCAGACCAGCTA-3′. The assays were performed in triplicate, and relative expression levels were calculated using the delta Ct method. Data were visualized using GraphPad Prism v5.0.

## Supplementary Material

dkaf121_Supplementary_Data

## Figures and Tables

**Table 1.** Antimicrobial susceptibility of the SME-6—producing *S. ureilytica* strain X47

Antibiotics	Routine diagnostic		Confirmatory AST					
	Vitek MIC (mg/L)	Gradient strips MIC	Vitek MIC, pre-incubated overnight at 37°C (mg/L)	Vitek MIC, pre-incubated overnight at 30°C (mg/L)	DD at 37°C (mm)	DD at 30°C (mm)	BMD MIC at 37°C (mg/L)	BMD MIC at 30°C (mg/L)
Ampicillin	**≥32**		**≥32**	**≥32**				
Piperacillin	≤4		≤4	≤4	26	22	≤8	≤8
Piperacillin-tazobactam	≤4		≤4	≤4	29	27	≤4	≤4
Cefotaxime	≤0.25		≤0.25	≤0.25			≤1	≤1
Ceftazidime	≤0.12		≤0.12	≤0.12	25	26	≤1	≤1
Imipenem	**≥16**	**≥32**	**8**	**8**	27	**14**	≤1	**>8**
Meropenem	**≥16**	**32**	≤0.5	**≥16**	26	**10**	≤0.125	**>16**
Meropenem-vaborbactam							≤0.12	0.25
Ciprofloxacin	≤0.06		≤0.06	≤0.06			≤0.25	≤0.25
Gentamicin	≤1		≤1	≤1				
Trimethoprim-sulfamethoxazole	≤20		≤20	≤20			≤1/19	≤1/19

Results in bold are considered resistant as defined by EUCAST.

AST, antimicrobial susceptibility testing; DD, disc diameter; BMD, broth microdilution.

**Figure 1.**
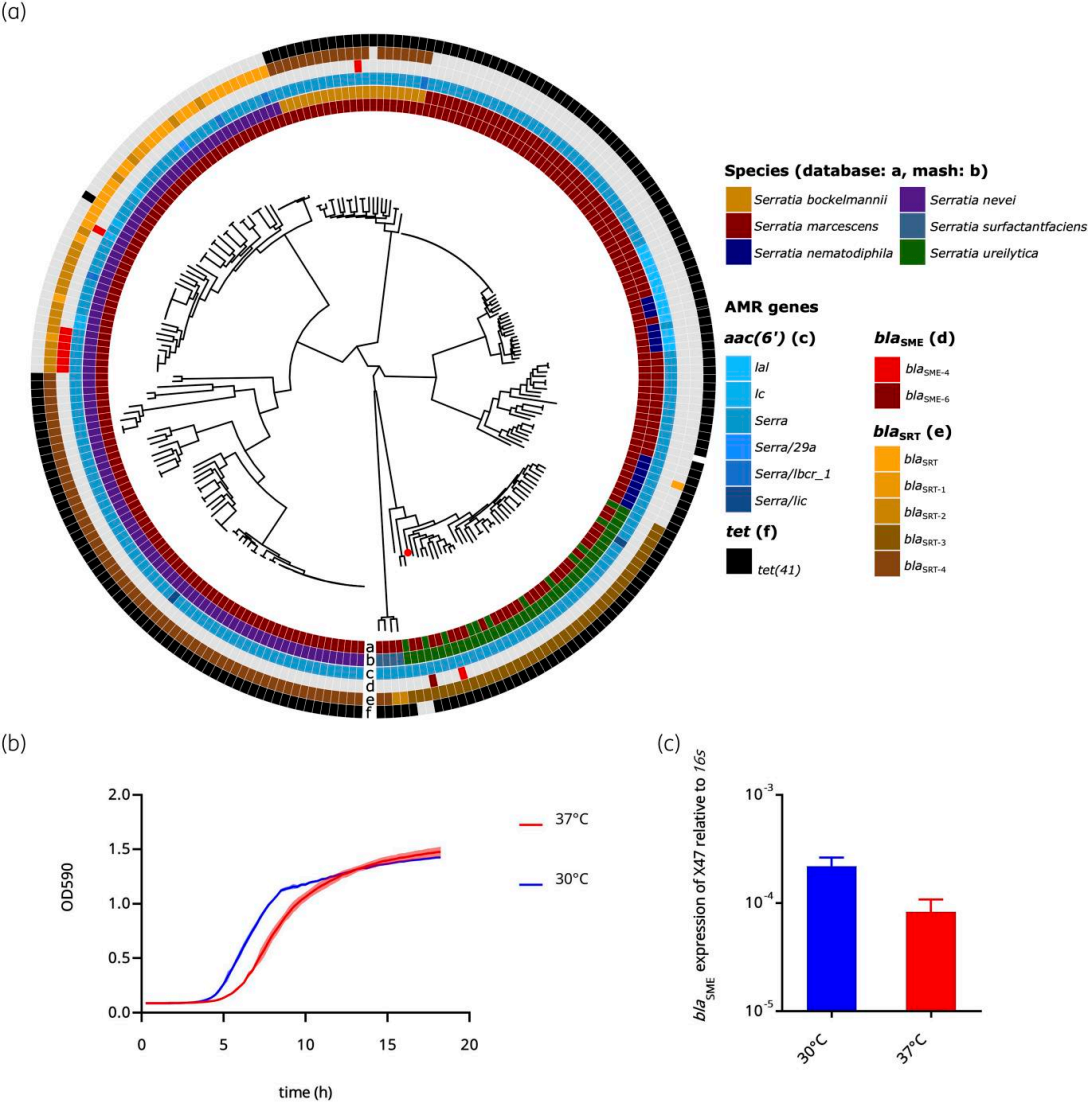
(a) Phylogenic distribution of our isolate compared with publicly available genomes. Phylogeny is based on the SNP distribution within the core-genome (2118 genes). The two first inner circle represent the taxonomy obtained from the NCBI/Refseq database (a) and the re-assigned taxonomy based on mash alignment with representative sequences of each species (b). The four outer circles show the presence and variants of the resistance genes *aac(6*′*)* (c), *bla*_SME_ (d), *bla*_SRT_ (e) and *tet* (f) (b) Growth curves of *S. ureilytica* strain X47 at 37°C and 30°C in antibiotic-free Mueller–Hinton broth. The line and the corresponding shaded areas are mean ± standard deviation for the replicates. (c) *bla*_SME_ mRNA expression of the *S. ureilytica* strain X47 after incubation to mid-log phase at 37°C and 30°C. Relative SME production in each condition were normalized to 16S mRNA expression. Mean values were calculated after three repeats. Error bars indicate standard deviation.

## Data Availability

The *bla*_SME-6_ sequence has been deposited in GenBank under accession number PQ361319.

## Results and discussion

Species identification by MALDI-TOF MS initially classified the isolate as *S. marcescens*. Initial routine diagnostics revealed the isolate to be highly resistant to both imipenem and meropenem (MIC > 32 mg/L), while remaining susceptible to extended-spectrum cephalosporins and piperacillin (Table 1). Carbapenem resistance was further confirmed by an MIC gradient test. The meropenem disk inactivation assay (CIM) exhibited meropenem degradation, which suggested the presence of a carbapenemase.

To elucidate the mechanism of carbapenem resistance, WGS was performed. WGS identified the isolate as *Serratia ureilytica*, a species often misclassified as *S. marcescens* (Figure 1a). Bioinformatic analysis uncovered a novel *bla*_SME_ variant, which was assigned as *bla*_SME-6_ after uploading the complete sequence to the NCBI Genbank database (accession number PQ361319). This gene shared close homology with the known SME variant *bla*_SME-2_ but differed by two amino acid substitutions: G117R and G147E (Figure S1, available as Supplementary data at *JAC* Online). Additionally, three other antimicrobial resistance genes were identified: *aac(6*′*)_serra*, *tet(41)*, and *bla*_SRT-3_, all of which are commonly found in *S. ureilytica* (Figure 1a).

The resistance phenotype was consistent with previous reports on SME carbapenemases, which typically confer resistance to carbapenems while sparing extended-spectrum cephalosporins and piperacillin-tazobactam.^3^ However, additional AST testing with BMD and disc diffusion revealed a temperature-dependent resistance profile. The strain was susceptible to both carbapenems at 37°C in BMD, while the CIM result remained positive at 37°C as in the routine diagnostics. Therefore, given the growth preference of *Serratia* spp. at 30°C,^23,24^ we investigated whether incubation temperature might influence the resistance phenotype and found that the strain was resistant to both carbapenems when incubated at 30°C (Table 1). The addition of vaborbactam to meropenem restored susceptibility at 30°C.

As the Vitek system’s standard incubation is at 37°C, we repeated the Vitek testing after pre-incubating the strain overnight at both temperatures. Notably, meropenem resistance was only observed after pre-incubation at 30°C, whereas imipenem was resistant at both temperatures (Table 1). This suggests that pre-incubation at 30°C may have increased basal *bla*_SME_ expression, potentially resulting in greater enzyme availability at the time of Vitek testing and subsequently higher meropenem MICs. The reason for the higher imipenem MICs in Vitek compared with BMD remains unclear, but might be due to methodological differences as Vitek estimates MICs by comparing growth patterns rather than directly measuring bacterial growth at specific antibiotic concentrations. Furthermore, SME-2, the closest phylogenetic relative of SME-6, has been reported to have significantly higher k_cat_ and relative k_cat_ values for imipenem compared with meropenem, indicating a higher hydrolytic activity against imipenem.^6^ This suggests that even lower levels of basal SME-6 expression may have been sufficient to confer imipenem resistance in Vitek, while the meropenem MIC at 37°C remained unaffected.

Growth curve analysis indicated that incubation temperature slightly influenced bacterial growth, with a longer lag phase observed at 37°C compared with 30°C—approximately 1.5 h (Figure 1b). This finding suggests that higher temperatures may delay initial bacterial growth and/or the expression of temperature-sensitive enzymes, potentially enhancing carbapenem efficacy against the strain. In support of this finding, RT-qPCR revealed that *bla*_SME_ mRNA expression was approximately 3-fold higher at 30°C compared with 37°C (Figure 1c). These results suggest that both faster bacterial growth and increased SME carbapenemase expression at lower temperatures are likely to contribute to the observed carbapenem resistance phenotype at 30°C.

A plausible explanation for the initial detection of carbapenem resistance during routine diagnostics is the effect of ambient summer temperatures, which often approach 30°C. The isolate was likely to have been exposed to a high ambient temperature prior to testing with Vitek, allowing for sufficient bacterial growth and/or *bla*_SME_ expression to facilitate accurate detection of resistance. Temperature has been previously implicated in the regulation of various genes in *Serratia marcescens*, including those involved in virulence factor activity, enhanced bacterial survival, and antimicrobial resistance determinants such as the *ompF* porin gene.^24–27^ However, to our knowledge, the influence of temperature on beta-lactamase activity has not been previously documented.

According to the EUCAST guidelines for resistance mechanism detection, the isolate could not be identified as resistant by BMD or disc diffusion methods. However, the imipenem MIC in Vitek remained in the resistant range even when pre-incubated at 37°C. Therefore, although meropenem showed high susceptibility, resistance could still have been suspected based on the Vitek imipenem MIC. In such cases involving *Serratia* spp., additional AST at different temperatures may be performed to investigate the temperature-dependent growth preferences and gene expression.

In conclusion, we report the identification of a novel SME-type carbapenemase, SME-6, in *Serratia ureilytica* isolated from a hospital in Germany. To the best of our knowledge, this is the first documented detection of an SME-type carbapenemase in Germany. Notably, the resistance phenotype was temperature dependent, with carbapenem resistance observed exclusively at 30°C. This finding suggests that SME-6 may be more common than previously recognized and may be underdiagnosed using standard diagnostic protocols. However, it is unclear whether this temperature-dependent resistance is clinically relevant in the human body, where the temperature is 37°C. Further studies are needed to determine whether SME-6 can confer carbapenem resistance under physiological conditions. Modifications to standard AST methods, such as incubation at different temperatures, may help to improve the detection of temperature-sensitive resistance mechanisms in *Serratia* species.

## Supplementary data

Figure S1 is available as Supplementary data at *JAC* Online.
