# Supplementary figures and images for: Detection of a novel SME-6 Carbapenemase in Serratia ureilytica in Germany
Source: J Antimicrob Chemother. 2025 Apr 12;80(6):1682–6. doi: 10.1093/jac/dkaf121 (PMC12129577; doi:10.1093/jac/dkaf121)

**Supplementary Figure S1. A phylogeny of known SME based on their amino acid sequences.**

**
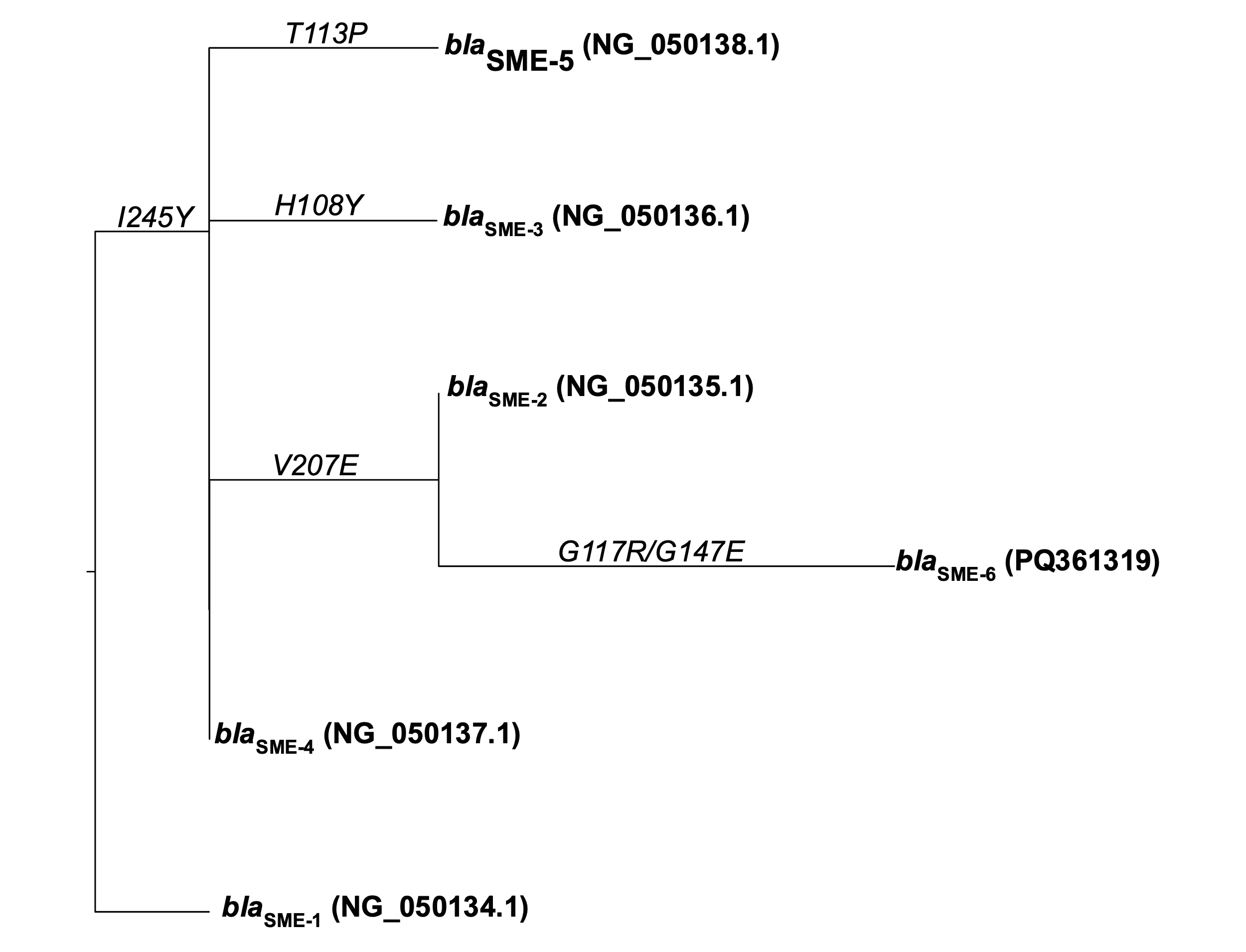
**

Supplement: dkaf121_Supplementary_Data [file dkaf121_supplementary_data.docx]
